# Supplementary material for: Between-Sex Differences in Risk Factors for Cardiovascular Disease among Patients with Myocardial Infarction—A Systematic Review
Source: J Clin Med. 2023 Aug 7;12(15):5163. doi: 10.3390/jcm12155163 (PMC10420061; doi:10.3390/jcm12155163)
Supplement: Supplementary file 1 [file jcm-12-05163-s001.zip › File S1.pdf]

MEDLINE search

(myocardial infarct*[TI] OR acute coronar*[TI] OR STEMI[TI] OR NONSTEMI[TI] OR NSTEMI[TI] OR ACS[TI] OR Acute Coronary Syndrome[TI]) AND (male[TI] OR female[TI] OR women[TI] OR men[TI] OR woman[TI] OR man [TI] OR sex [TI]) AND (prospective[abs] OR retrospective [abs] OR case-control*[abs] OR cohort [abs] OR cross-sectional [abs] OR observational [abs])

EMBASE search

#24 ((myocardial AND infarc$:ti) OR (acute AND coronar$:ti) OR (acute AND coronar$:ti) OR stemi:ti OR nonstemi:ti OR nstemi:ti OR acs:ti OR (acute AND coronary AND syndrome:ti)) AND (male:ti OR female:ti OR wom*n:ti OR (man OR men:ti) OR sex:ti) AND (prospective:ab OR retrospective:ab OR case*control:ab OR cohort:ab OR cross*sectional:ab OR observational:ab) AND [english]/lim AND [01-01-2012]/sd NOT [01-10-2022]/sd

#23 ((myocardial AND infarc$:ti) OR (acute AND coronar$:ti) OR (acute AND coronar$:ti) OR stemi:ti OR nonstemi:ti OR nstemi:ti OR acs:ti OR (acute AND coronary AND syndrome:ti)) AND (male:ti OR female:ti OR wom*n:ti OR (man OR men:ti) OR sex:ti) AND (prospective:ab OR retrospective:ab OR case*control:ab OR cohort:ab OR cross*sectional:ab OR observational:ab)

#22 prospective:ab OR retrospective:ab OR case*control:ab OR cohort:ab OR cross*sectional:ab OR observational:ab

#21 observational:ab

#20 cross*sectional:ab

#19 cohort:ab

#18 case*control:ab

#17 retrospective:ab

#16 prospective:ab

#15 male:ti OR female:ti OR wom*n:ti OR (man OR men:ti) OR sex:ti

#14 sex:ti

#13 man OR men:ti

#12 wom*n:ti

#11 female:ti

#10 male:ti

#9 (myocardial AND infarc$:ti) OR (acute AND coronar$:ti) OR (acute AND coronar$:ti) OR stemi:ti OR nonstemi:ti OR nstemi:ti OR acs:ti OR (acute AND coronary AND syndrome:ti)

#8 acute AND coronary AND syndrome:ti

#7 acs:ti

#6 nstemi:ti

#5 nonstemi:ti

#4 stemi:ti

#3 acute AND coronar$:ti

#2 acute AND coronar$:ti

#1 myocardial AND infarc$:ti

CENTRAL

#1 (acute coronar*):ti
#2 (myocardial infarct*):ti
#3 (STEMI):ti
#4 (NONSTEMI):ti
#5 (NSTEMI):ti
#6 (ACS):ti
#7 (acute coronary syndrome):ti
#8 #1 or #2 or #3 or #4 or #5 or #6 or #7
#9 (male):ti
#10 (female):ti
#11 (women):ti
#12 (men):ti
#13 (woman):ti
#14 (man):ti
#15 (sex):ti
#16 #9 or #10 or #11 or #12 or #13 or #14 or #15
#17 #8 and #16
