# Supplementary material for: Between-Sex Differences in Risk Factors for Cardiovascular Disease among Patients with Myocardial Infarction—A Systematic Review
Source: J Clin Med. 2023 Aug 7;12(15):5163. doi: 10.3390/jcm12155163 (PMC10420061; doi:10.3390/jcm12155163)
Supplement: Supplementary file 1 [file jcm-12-05163-s001.zip › jcm-2479190-Table S1.pdf]

Study	Selection	Comparability	Outcome	Total	Quality of Study
Ahmadi et al. 2015	***	**	**	7	High
Asleh et al. (2022)	****	**	***	9	High
Bajaj et al. (2016)	***	**	**	7	High
Baumann et al. (2016)	****	**	***	9	High
Canto et al. (2012)	****	**	***	9	High
Dreyer et al. (2013)	****	**	***	9	High
Gardarsdottir et al. (2022)	****	**	***	9	High
Khraishah et al. (2021)	***	**	**	7	High
Krishnamurthy et al. (2019)	****	**	***	9	High
Leurent et al. (2014)	***	**	***	8	High
Nguyen et al. (2014)	****	**	**	8	High
Ortalani et al. (2013)	***	**	***	8	High
Radovanovic et al. (2012)	****	**	***	9	High
Redfors et al. (2015)	****	**	***	9	High
Roque et al. (2020)	***	**	*	6	Medium
Strömbäck et al. (2017)	****	**	***	9	High
Velders et al. (2013)	****	**	***	9	High
Result of Risk of Bias Assessment as per Newcastle-Ottawa Scale [24].					

Each star represents one point within each respective category of the Newcastle-Ottawa quality assessment scale for cohort studies. The star system is recommended by the original assessment scale itself. There are a maximum of 4 stars available for the selection category, 2 for comparability and 3 for outcome, yielding a maximum score of 9. The original template assessment scale itself describes the requirements for each star in each category [24].
